# Genome-wide identification of sucrose non-fermenting-1-related protein kinase genes in maize and their responses to abiotic stresses

**DOI:** 10.3389/fpls.2022.1087839

**Published:** 2022-12-22

**Authors:** Xue Feng, Quan Meng, Jianbin Zeng, Qian Yu, Dengan Xu, Xuehuan Dai, Lei Ge, Wujun Ma, Wenxing Liu

**Affiliations:** ^1^ College of Agronomy, Qingdao Agricultural University, Qingdao, China; ^2^ State Agricultural Biotechnology Centre, College of Science, Health, Engineering and Education, Murdoch University, Perth, WA, Australia; ^3^ The Key Laboratory of the Plant Development and Environmental Adaptation Biology, Ministry of Education, School of Life Sciences, Shandong University, Qingdao, Shandong, China

**Keywords:** maize (*Zea mays*), SnRK, genome-wide, expression patterns, climate-resilience

## Abstract

**Introduction:**

Protein kinases play an important role in plants in response to environmental changes through signal transduction. As a large family of protein kinases, sucrose non-fermenting-1 (SNF1)-related kinases (SnRKs) were found and functionally verified in many plants. Nevertheless, little is known about the *SnRK* family of *Zea mays*.

**Methods:**

Evolutionary relationships, chromosome locations, gene structures, conserved motifs, and cis-elements in promoter regions were systematically analyzed. Besides, tissue-specific and stress-induced expression patterns of *ZmSnRKs* were determined. Finally, functional regulatory networks between *ZmSnRKs* and other proteins or miRNAs were constructed.

**Results and Discussion:**

In total, 60 *SnRK* genes located on 10 chromosomes were discovered in maize. *ZmSnRKs* were classified into three subfamilies (*ZmSnRK1*, *ZmSnRK2*, and *ZmSnRK3*), consisting of 4, 14, and 42 genes, respectively. Gene structure analysis showed that 33 of the 42 *ZmSnRK3* genes contained only one exon. Most *ZmSnRK* genes contained at least one ABRE, MBS, and LTR cis-element and a few *ZmSnRK* genes had AuxRR-core, P-box, MBSI, and SARE ciselements in their promoter regions. The Ka:Ks ratio of 22 paralogous *ZmSnRK* gene pairs revealed that the *ZmSnRK* gene family had experienced a purifying selection. Meanwhile, we analyzed the expression profiles of *ZmSnRKs*, and they exhibited significant differences in various tissues and abiotic stresses. In addition, A total of eight ZmPP2Cs, which can interact with *ZmSnRK* proteins, and 46 miRNAs, which can target 24 *ZmSnRKs*, were identified. Generally, these results provide valuable information for further function verification of *ZmSnRKs*, and improve our understanding of the role of *ZmSnRKs* in the climate resilience of maize.

## Introduction

Terrestrial plants must cope with environmental changes including drought; excess salt, heat, cold, and toxic metals; and nutrient deficiency (Zhu, 2016). Protein phosphatase and kinase-regulated signal transduction play a vital role in climate resilience in plants (Hunter, 1995; Cohen, 1988). In recent years, several protein kinases have been identified, including receptor protein kinase (RLK) (Tör et al., 2009), mitogen-activated protein kinase (MAPK) (Zhang and Zhang, 2022), calcium-dependent protein kinase (CDPK) (Bender et al., 2018), and sucrose non-fermenting-1 (SNF1)-related kinase (SnRK) (Maszkowska et al., 2021).

SnRKs in plants have a high sequence identity with SNF1 of fungi and AMP-activated protein kinase (AMPK) of mammals (Halford and Hardie, 1998). The first plant SnRK genes were identified in *Secale cereale*, and showed 48% amino acid homology with SNF1 and AMPK (Alderson et al., 1991). In plants, the SnRK family has expanded and diverged into three subfamilies (SnRK1, SnRK2, and SnRK3) (Hrabak et al., 2003). The N-terminal of three SnRK subfamilies have a conserved domain with serine/threonine protein kinase, while the C-terminal displayed variation. In detail, SnRK1s were most similar to SNF1 and AMPK in terms of structure and function. SnRK2s contain acidic amino acids in the C-terminal domain, either Glu or Asp (Kulik et al., 2011). SnRK3s were widely recognized as CIPKs (CBL-interacting protein kinases) (Tominaga et al., 2010), which have two conserved domains in the C-terminal: NAF and PPI (Masaru et al., 2003; Albrecht et al., 2014).

SnRK1s are evolutionarily conserved energy-sensing protein kinases in plants. When energy supplies become limited, SnRK1s can activate transcriptional regulatory networks and maintain cellular energy homeostasis (Wurzinger et al., 2018; Wang et al., 2021). Therefore, SnRK1s were known as conserved energy sensors. For example, Wang et al. (2021) reported that the α-subunit of OsSnRK1 could phosphorylate JMJ705 to stimulate its demethylase activity, thus targeting and activating a series of low-energy stress-responsive transcription factors. SnRK1 phosphorylates the transcription factor basic leucine zipper63 (bZIP63), which could directly bind the promoter of auxin response factor19 (ARF19) and activate gene expression to mediate lateral root development (Muralidhara et al., 2021). In peach fruit, SnRK1 could phosphorylate sorbitol dehydrogenase and lead to sugar accumulation (Yu et al., 2021). In maize, SnRK1 could coordinate carbon and nitrogen absorption in seeds by phosphorylating E3 ubiquitin ligase ZmRFWD3 (Li et al., 2020).

SnRK2s are much smaller than SnRK1 and are associated with the ABA signal transduction pathway (Cutler et al., 2010; Maszkowska et al., 2021). In rice, a mutation of *OsSAPK8*, a member of SnRK2, exhibited sensitivity to diverse abiotic stresses at the vegetative stages, such as drought stresses, excess of salt, and low temperature (Zhong et al., 2020). In Arabidopsis, overexpression of *AtSnRK2.8* showed higher resistance to drought. *AtSnRK2.10* was induced by salt stress and was involved in salt tolerance through increasing photosynthesis and protecting against oxidative damage (Mazur et al., 2021). The activity of SnRK2.4 was significantly increased in roots after 30s of salt exposure, but it disappeared 1h later (Mcloughlin et al., 2012). In rice, SnRK2.6/OST1 was immediately activated in an ABA non-responsive pathway to elevate low temperature adaptation (Ding et al., 2015).

SnRK3s could combine with Ca^2+^-dependent CBL to regulate ion transport and enhance environmental adaption in plants, especially in abiotic stress. For instance, AtCIPK24 protein could interact with AtCBL4 to phosphorylate and activate plasma membrane H^+^/Na^+^ antiporter (SOS1) in a Ca^2+^-responsive manner in Arabidopsis (Qiu et al., 2002). In rice, the expression of *OsCIPK31* was up-regulated under several abiotic stresses during germination and seedling growth (Piao et al., 2010). Ma et al. (2021) indicated that CaCIPK3 positively regulated drought tolerance by the CBL-CIPK network, thus mediating MeJA signaling and the antioxidant defense system. Moreover, the OsCBL1-OsCIPK23 complex could enhance Os-AKT1-mediated K^+^ uptake (Li et al., 2014).

Maize is the most commonly cultivated food crop globally. However, research concerning the role of *SnRK* genes is still rare in maize. With the rapid progress of molecular biology and the whole-genome sequencing of maize (Myburg et al., 2014), it becomes possible for us to have a comprehensive understanding of *ZmSnRK* gene families. Here, 60 candidate *ZmSnRK* genes were discovered by bioinformatic analysis. Then, evolutionary relationships, chromosome locations, gene structures, conserved motifs, and cis-elements in promoter regions were systematically analyzed. Besides, tissue-specific and stress-induced expression patterns of *ZmSnRKs* were determined. Finally, functional regulatory networks between ZmSnRKs and other proteins or miRNAs were constructed. All these results offer valuable information for further function verification of *ZmSnRKs* and help to better comprehend the role of *ZmSnRKs* in the climate resilience of maize.

## Materials and methods

### Identification and general characterization analysis of ZmSnRK proteins

ZmSnRK proteins were searched using query probes of AtSnRKs and OsSnRKs by BLASTP. We used Zm-B73 V5.0 database from the MaizeGDB (https://maizegdb.org/) to conduct BLASP. SnRKs in Arabidopsis and rice were extracted from ensemble plants (https://plants.ensembl.org/). Meanwhile, the HMMER 3.0 was utilized to search the ZmSnRKs protein sequence according to the Hidden Markov Model (HMM) files of SnRKs acquired from the Pfam database (http://pfam.xfam.org/). The SMART database was used to reconfirm sequences (Letunic et al., 2012). The molecular weights and isoelectric points of the proteins were analyzed through the ExPASy tool (www.expasy.org/tools/). Subcellular localization was also predicted through WoLF PSORT (https://wolfpsort.hgc.jp/).

### Phylogenetic analysis and classification of ZmSnRKs

Multiple homology alignment of 60 ZmSnRK non-redundant amino acid sequences and SnRK protein sequences from *Arabidopsis thaliana*, *Oryza sativa*, and *Zea mays* were conducted by ClustalW with default parameters (Larkin et al., 2007). Then, we used MEGA 7.0 to construct a phylogenetic tree *via* neighbor-joining (NJ) method (Kumar et al., 2016), with the following parameters: Poisson model, pairwise deletion, and 1,000 bootstrap replications.

### Chromosome localization and collinearity analysis of *ZmSnRKs*

Chromosome localization of *ZmSnRKs* was conducted using MapChart v. 2.3 Software (Voorrips, 2002). The collinearity of the orthologous *SnRK* genes between *Zea mays* and other species, such as *Arabidopsis thaliana*, *Oryza sativa*, and *Hordeum vulgare*, was determined through MCScanX software (Wang et al., 2012). The non-synonymous substitution rate (Ka) and synonymous substitution rate (Ks) of ZmSnRKs in the gene collinearity were calculated by using the ParaAT tool (Zhang et al., 2012). The Ka/Ks values were calculated by Calculator 2.0 software (Wang et al., 2010). The gene duplicated time was assessed based on Ks/2λ (Wang et al., 2010), where λ = 1.5 × 10^−8^.

### Analysis of gene structure and motifs of *ZmSnRKs*

The exon and intron arrangement of *ZmSnRK* genes was determined *via* the Gene Structure Display Server (GSDS) tool (http://gsds.cbi.pku.edu.cn/) (Hu et al., 2015). The conserved motif sequences of ZmSnRK proteins were identified using Multiple Expectation Maximization for Motif Elicitation (MEME) online program (http://meme.sdsc.edu/meme/itro.html) (Bailey et al., 2009). InterProScan (www.ebi.ac.uk/Tools/pfa/iprscan/) was utilized to annotate the motifs (Jones et al., 2014). The gene structures and conserved motifs of *ZmSnRKs* were visualized through TBtools (Chen et al., 2020).

### Predication of cis-acting elements in the promoter regions of *ZmSnRKs*

Promoter sequences (-1500 bp) of *ZmSnRK* genes were obtained from the maize B73 genome. The cis-elements were determined by PlantCARE software (http://bioinformatics.psb.ugent.be/webtools/plantcare/html/) (Magali et al., 2002). We then use TBtools to visualize the cis-elements (Chen et al., 2020) associated with abiotic stress, including abscisic acid response components ABRE, drought response components MBS, auxin response components AuxRR-core, flavonoid biosynthetic genes regulation components MBSI, gibberellin-response components P-box, salicylic acid response components SARE, and low-temperature response compositions LTR.

### Modeling of 3D structures of ZmSnRKs

The 3D structures of ZmSnRK proteins were predicted using Swiss-Model (https://swissmodel.expasy.org/interactive/) (Arnold et al., 2006). Then, the quality of 3D protein structure was assessed *via* SAVES server (http://nihserver.mbi.ucla.edu/SAVES/).

### Expression profiles and interaction networks analysis of ZmSnRKs

The specific expression patterns of *SnRKs* from various tissues and under abiotic stress in the maize B73 were obtained from qTeller in MaizeGDB (https://qteller.maizegdb.org/) (Opitz et al., 2014; Stelpflug et al., 2016). We used transcript per million (TPM) reads mapped to represent the gene expression values. The genes with |log2 ratio|≥1 were recognized as differentially expressed genes (DEGs). A heatmap was conducted by TBtool to exhibit expression levels in various tissues (log_2_^(TPM+1)^)and different abiotic stresses (log_2_^fold change^). Moreover, the protein interactions were predicted by the Search Tool for the Retrieval of Interacting Genes (STRING) (http://string-db.org/cgi) (Damian et al., 2021). We used Cytoscape software to create interaction networks (Shannon et al., 2003). A low confidence (0.15) was used as the “minimum required interaction score” when analyzing protein interaction within ZmSnRKs, and high confidence was used as “maximum required interaction score” when analyzing protein interaction between ZmSnRKs and ZmPP2Cs.

### Prediction of microRNAs-*ZmSnRKs* regulatory networks

The microRNA (miRNA)-*ZmSnRK* regulatory networks were predicted on the basis of the previous methods (Su et al., 2021). In brief, the *ZmSnRK*-targeting miRNAs were predicted with coding sequences of *ZmSnRKs* through the psRNATarget server (http://plantgrn.noble.org/psRNATarget/home) under default parameters except for that penalty for G:U pair was 1 and mismatches allowed in seed region was 0. The miRNA-targeted sites were those highly complementary to coding sequences of *ZmSnRKs*. The interaction networks were created using the Cytoscape V3.9.1 software (https://cytoscape.org/download.html).

### Plant growth and treatment

The hydroponic experiment was conducted in a greenhouse at Qingdao Agricultural University, Qingdao, China. Maize inbred line B73 was used in this study. Seeds of maize were treated with 3% H_2_O_2_ for 10 min, and were rinsed seven times with distilled water. The seeds were sown in a controlled environment with a day/night temperature of 28/22°C on moist filter papers. After germination, uniform seedlings were transferred to 2 L pots containing 1.5 L of basic nutrient solution (BNS). On the seventh day after transplanting, PEG was added to the containers to form three treatments: BNS (control), BNS plus 10% PEG, and BNS plus 100 mM NaCl. The composition of BNS was (mg L*^−^* ^1^) MgSO_4_·7H_2_O, 160.21; K_2_SO_4_, 130.7; KCl, 7.455; KH_2_PO_4_, 34.02; Ca(NO_3_)_2_·4H_2_O, 472.3; Fe(III)-EDTA-Na, 36.7; MnSO_4_·H_2_O, 0.17; ZnSO_4_·7H_2_O, 0.29; CuSO_4_·5H_2_O, 0.025; (NH_4_)_6_Mo_7_O_24_·4H_2_O, 0.006; and HBO_3_, 0.062. The solution pH was adjusted to 5.8–6.0 with NaOH or HCl, as required. Plant leaves and stems were sampled on the seventh day after transplanting. Plant root samples for RNA isolation were collected 24h after treatment. All samples were stored at *−*80°C for downstream analysis.

### Quantitative RT−PCR validation

Quantitative real-time PCR (qRT-PCR) was performed by a QuantStudio3 PCR system (Thermo, USA). First-strand cDNA synthesis was performed using the TOROBlue^®^ qRT Premix with gDNA Eraser 2.0 (TOROIVD, China), followed by qRT-PCR using a TOROGreen^®^ qPCR Master Mix (TOROIVD, China) with *ZmActin* as a reference. The total PCR volume was 20 μl. The reaction process was 94°C denaturation for 1 min, followed by 40 cycles of 95°C for 10 s, 60°C for 30 s. Experiments were replicated three times with 2^-ΔΔCt^ relative quantification method. Primers were listed in **Table S14**.

### Statistical analysis

Statistical analyses were performed with a Processing System statistical software package using ANOVA followed by Duncan’s multiple range test.

## Results

### Identification and evolution of SnRKs in maize

In total, we identified 60 ZmSnRK proteins in the maize inbred line B73 genome. The detailed information in terms of genes and proteins can be seen in **Tables S1 and S2**. For instance, the amino acid length of 60 ZmSnRKs protein ranges from 215 to 653, and the molecular weight ranges from 24.7 to 72.3 kDa. All ZmSnRKs were predicted to be located in the nucleus, cytoplasm, or mitochondria.

**Figure 1:**
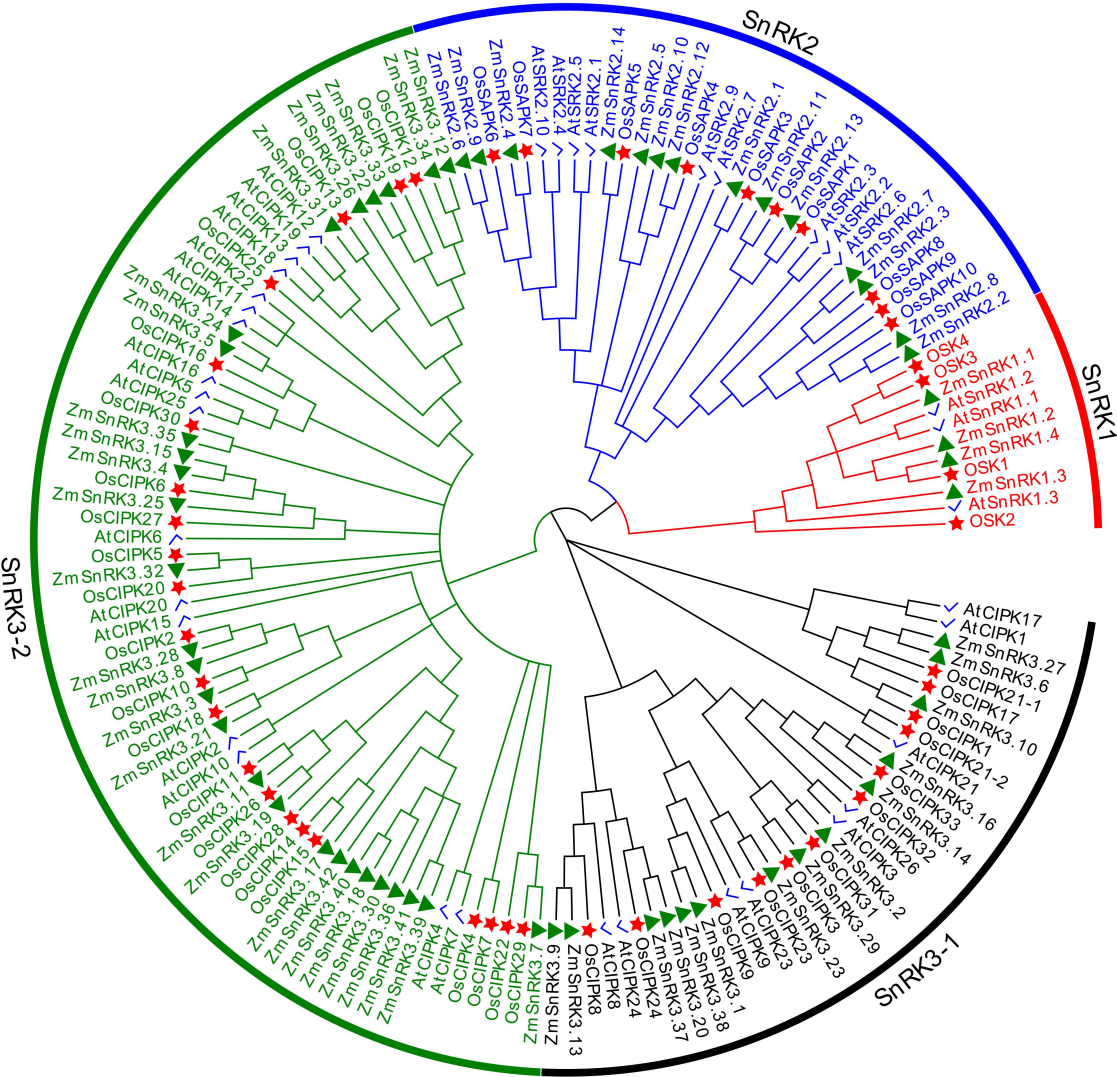
Phylogenetic tree of full-length ZmSnRK, AtSnRK and OsSnRK proteins. The different colored arcs indicate subfamilies of the SnRK proteins. Different colour shapes represent SnRKs from maize (▲), rice (☆), and Arabidopsis (√). The unrooted neighbor-joining phylogenetic tree was constructed using MEGA7 with full-length amino acid sequences and the bootstrap test replicate was set as 1,000 times.

**Figure 2:**
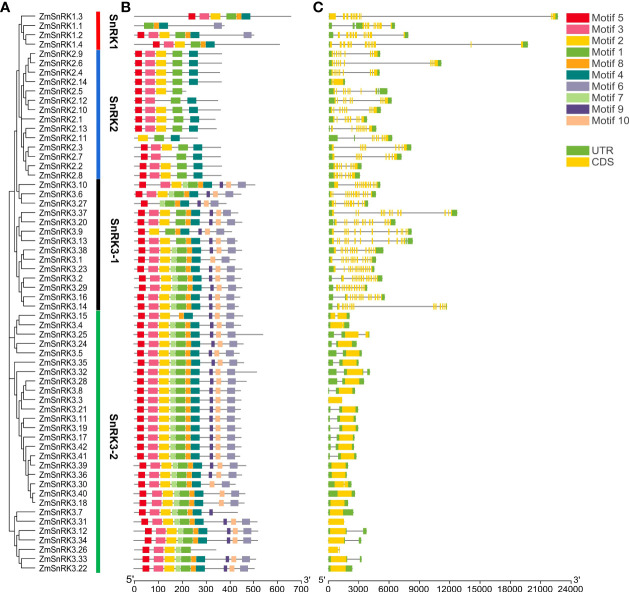
Phylogenetic relationships, architecture of conserved protein motifs and gene structure in *SnRK* genes from maize. **(A)** The phylogenetic tree was constructed based on the full-length sequences of maize SnRK proteins using MEGA 7 software. **(B)** The motif compositions of 60 ZmSnRK proteins. The motifs were identified using the MEME program. Boxes of different colors represent motifs 1 to 10. The length of the amino acid sequences can be estimated by the scale at the bottom. **(C)** Gene structures of 60 *ZmSnRK* genes. Yellow boxes represent exons, green boxes represent 5′ or 3′ untranslated regions (UTR), and black lines represent introns. The length of nucleotide sequences of exons/introns/UTRs can be estimated by the scale at the bottom.

To verify the evolutionary relations of SnRK proteins in angiosperms, we constructed a neighbor-joining tree using 47 rice SnRKs, 39 Arabidopsis SnRKs, and 60 maize SnRKs (**Figure 1**). The protein sequences in rice and Arabidopsis are listed in **Table S3**. Hrabak et al., (2003) reported that 39 AtSnRKs were classified into three subfamilies. Here, 60 ZmSnRKs were also clustered into three groups in accordance with phylogenetic analysis. In detail, four proteins containing Pkinase (PF00069), UBA (PF00627), and KA1 (PF02149) domains belong to the ZmSnRK1 subfamily; 14 proteins belong to the ZmSnRK2 subfamily, and the rest of 42 proteins containing Pkinase and NAF (PF03822) domains were clustered into the ZmSnRK3 subfamily (**Figure 2A**).

### Gene and protein structures in ZmSnRKs

The web server GSDS was used to understand the gene structure of *ZmSnRKs*, which had 1 to 14 exons unevenly. In detail, *SnRK1* subfamily genes contain 8–12 exons. *SnRK2* subfamily genes contain 7–9 exons, except *ZmSnRK2.2* and *ZmSnRK2.14* containing one exon. Interestingly, *ZmSnRK3* subfamily genes exhibit a large difference in the number of exons, ranging from 1 to 14 (**Figure 2C**). For example, half of the genes have only one exon, five genes have two exons, and another gene has 12–14 exons. Hence, *ZmSnRK3-1* and *ZmSnRK3-2* were classified in terms of the number of exons.

**Figure 3:**
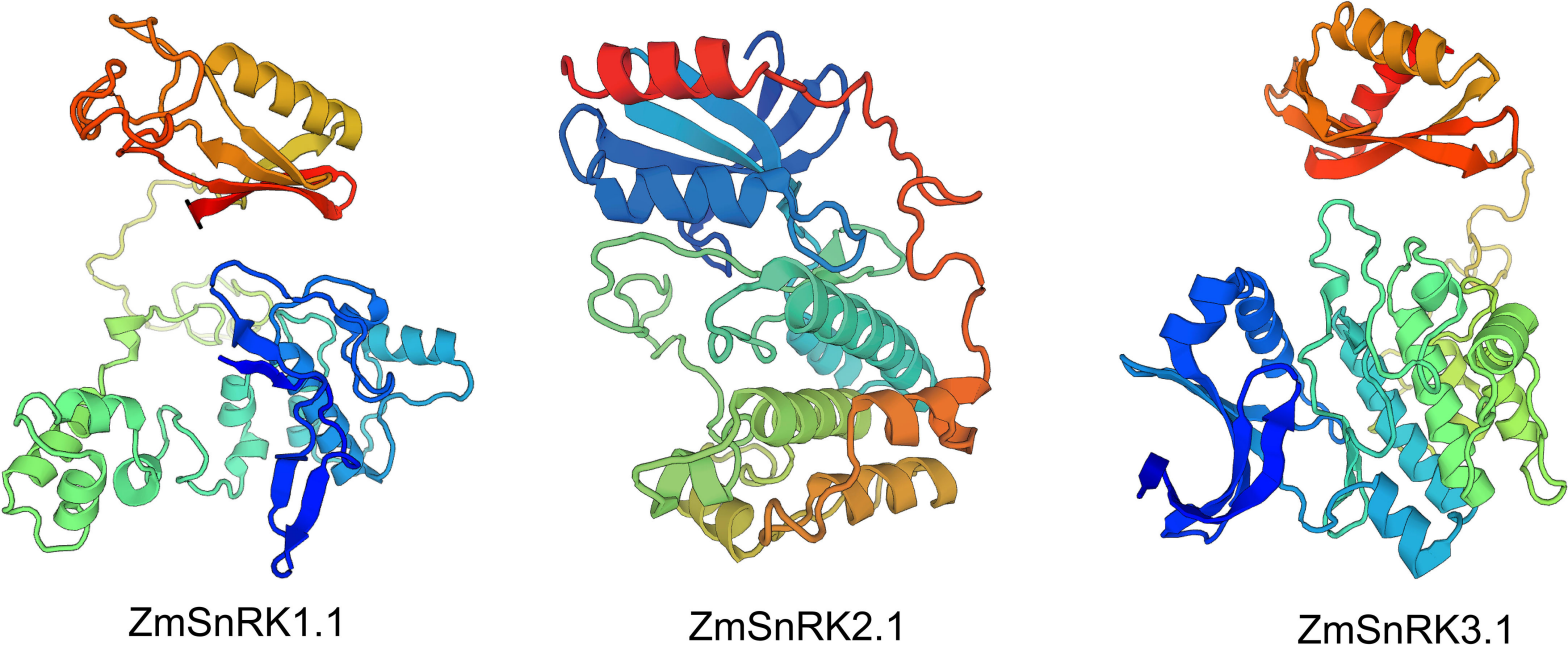
The 3D structure modeling of ZmSnRK proteins. The pymol software was used to create the structural image.

We used MEME to determine protein motifs. Generally, 10 conserved motifs were discovered in ZmSnRKs and named motifs 1–10 (**Figure 2B**; **Table S4**). Among them, the motifs ½/3/4/5 belong to phosphokinase domains. Almost all of ZmSnRKs have motifs 1, 2, 3, and 4, except ZmSnRK1.1 with motif 2/3/4, ZmSnRK3.27 and ZmSnRK2.7 with motif 1/3/4, ZmSnRK2.11 with motif 1/2/4, and ZmSnRK3.26 with motif 1/2/3. In addition, motif 6/7/9/10 exists exclusively in ZmSnRK3, except ZmSnRK1.1 and ZmSnRK1.4 containing motif 6. Moreover, motif 8 exists only in ZmSnRK1 and ZmSnRK3, except ZmSnRK3.26. In addition, 3D structures of ZmSnRK proteins were predicted and it showed that different ZmSnRKs own similar 3D structures in the N-terminal but disparate in the C-terminal (**Figure 3**).

### Chromosomal localization and duplication of *ZmSnRK*s

**Figure 4:**
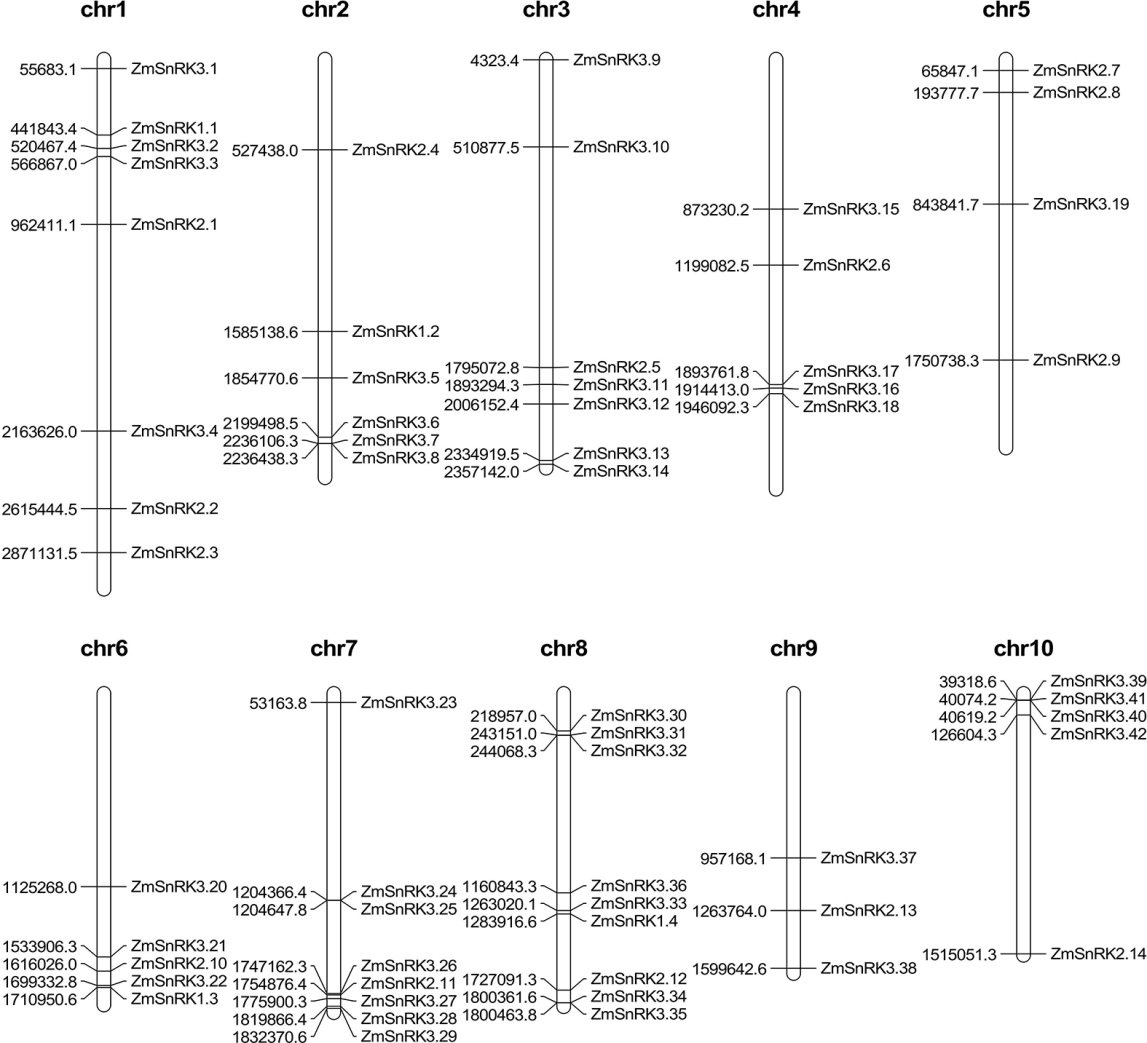
Distribution of *ZmSnRK* genes in maize chromosomes. The chromosome numbers are indicated at the top of each chromosome image.

**Figure 5:**
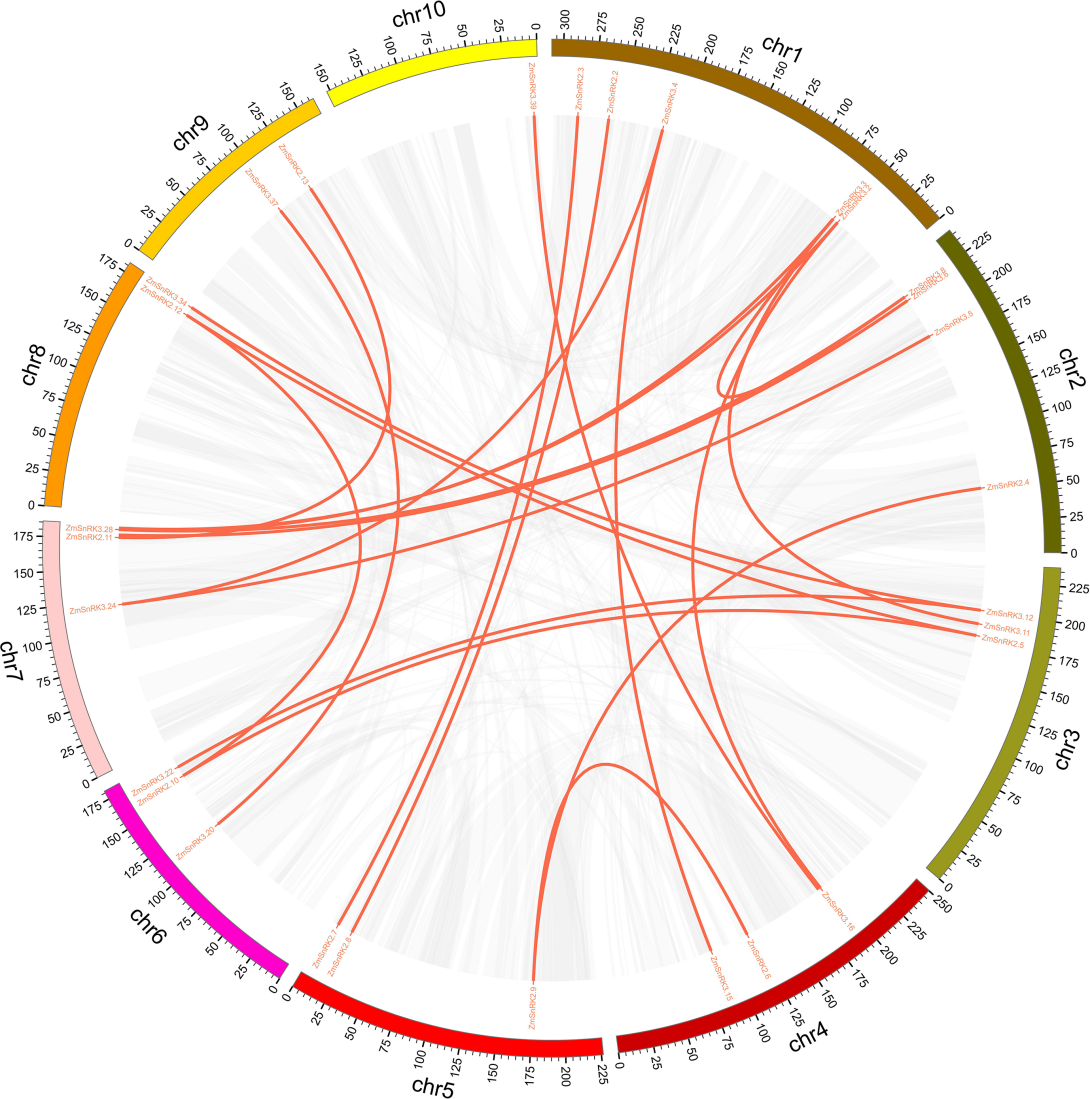
The synteny analysis of *ZmSnRK* family in maize. Gray lines indicate all synteny blocks in the maize genome. The genes linked by red lines represent homologues.

**Figure 6:**
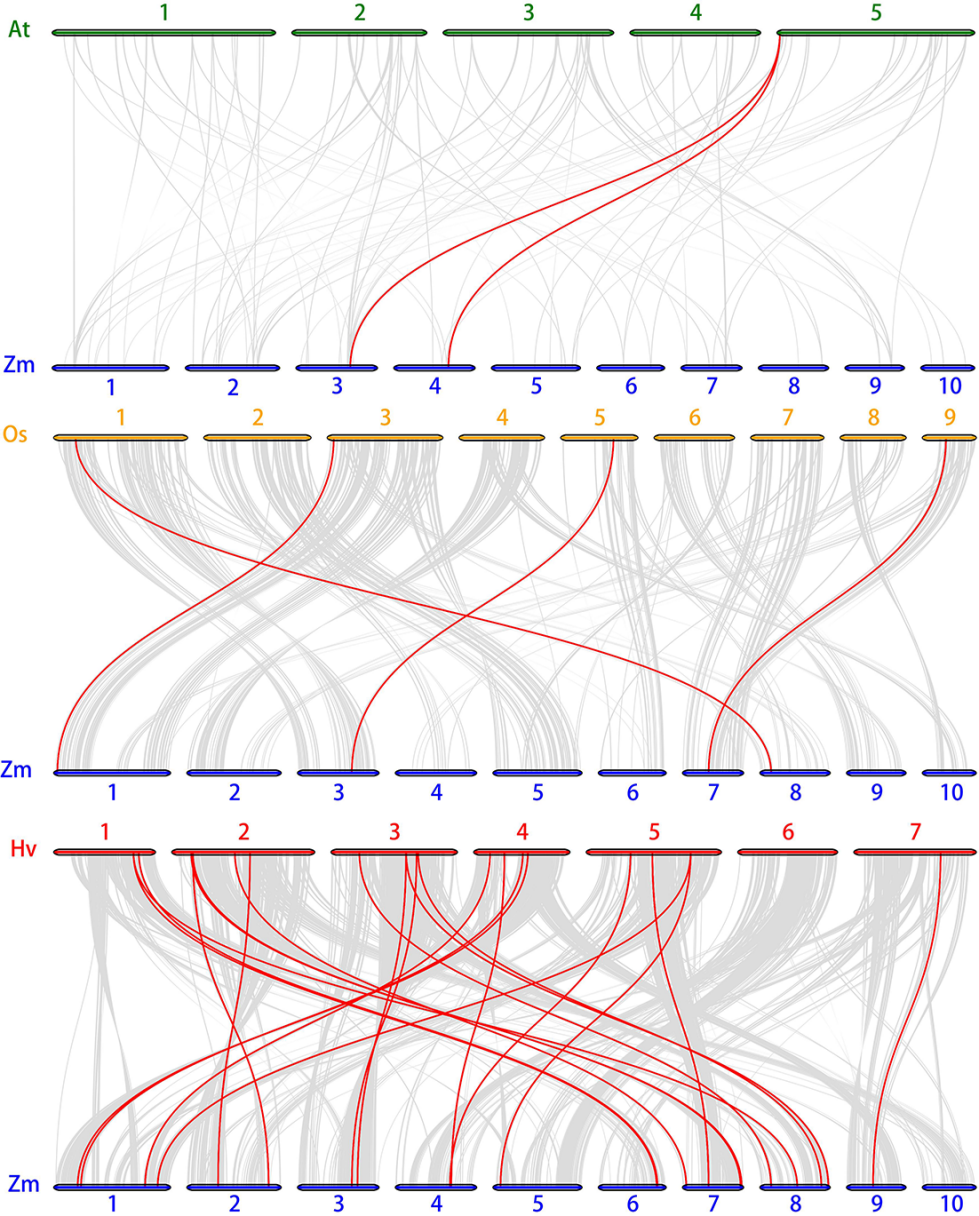
Synteny analysis of *SnRK* genes between maize, Arabidopsis, rice and barley. Gray lines: all collinear blocks within maize and other plant genomes. Red lines: the synteny of SnRK gene pairs. The specie names with the prefixes, Hv, Zm, At, and Os indicate barley, maize, Arabidopsis, and rice, respectively.

The chromosome location of *ZmSnRK* genes was analyzed and 60 *ZmSnRKs* were mapped to the maize genome (**Figure 4**). In detail, the gene members of the *ZmSnRK1* subfamily were localized in chromosomes 1, 2, 6, and 8. Both *ZmSnRK2* and *ZmSnRK3* subfamily genes were located in all chromosomes. We used the approach of BLAST and MCScanX to search for gene duplication. Finally, 22 segmental duplication events were detected in *ZmSnRKs*, and each gene pair was located on a distinct chromosome (**Figure 5**; **Table S5**). It demonstrated that most of *ZmSnRKs* were probably derived from gene duplication, and segmental duplication events could take a dominant role in the expansion of *ZmSnRK* genes in maize. Moreover, the syntenic relations of the *SnRK* gene families between maize genome, barley genome, Arabidopsis genome, and rice genome were also determined. The results showed that two *SnRK* gene pairs were found in maize and Arabidopsis, and four *SnRK* gene pairs were discovered in maize and rice (**Figure 6**; **Table S6, S7**). For instance, *AtCIPK15* showed colinearity with *ZmSnRK3.11* and *ZmSnRK3.17*. Furthermore, 24 *ZmSnRKs* exhibited syntenic relations with *SnRKs* in barley (**Figure 6**; **Table S8**).

To understand the evolutionary constraints acting on paralogous *SnRK* family genes, Ks value, Ka value, Ka : Ks ratio, and divergence time were calculated (**Table S5**). Most of the segmental duplication of *ZmSnRK* gene pairs displayed Ka/Ks ratios less than 1, except *ZmSnRK3.3/ZmSnRK3.8* and *ZmSnRK3.6/ZmSnRK3.27* with 2.69 and 1.02, respectively. The average value of *ZmSnRK3* (Ka/Ks = 0.56) was higher than *ZmSnRK2* gene pairs (Ka/Ks = 0.13). Divergence time was therefore estimated to occur between 4.304 Mya and 68.812 Mya ago. The above results implied that *ZmSnRK* gene families were likely to have suffered strong purifying selective pressure in the course of evolution.

### Cis-elements analysis in the promoters of *ZmSnRKs*

**Figure 7:**
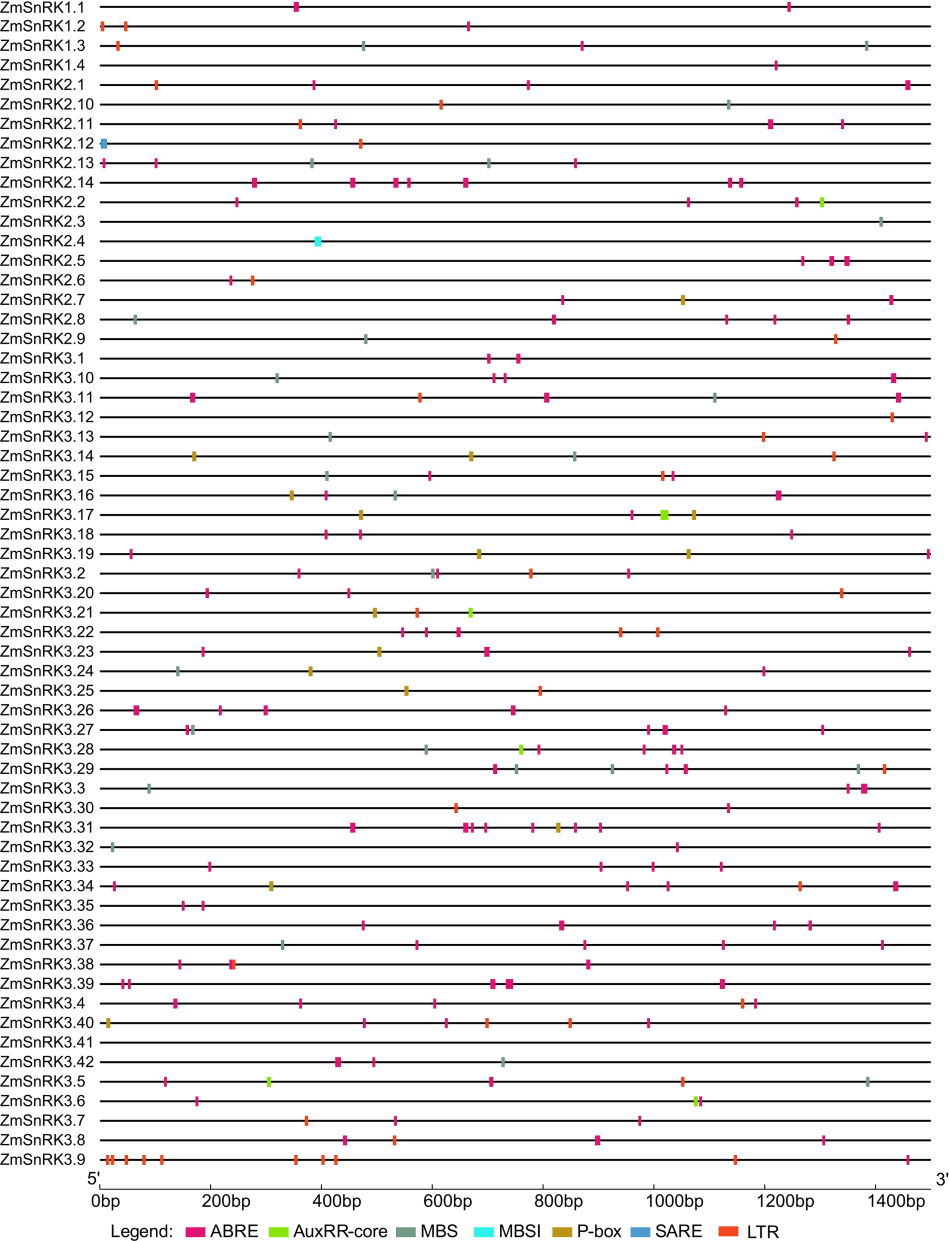
Predicted cis-regulatory elements in *ZmSnRK* promoters. Promoter sequences (about 1.5 kb) of 60 *ZmSnRK* genes were analyzed by PlantCARE. The upstream length to the translation starting site can be inferred according to the scale at the bottom.

Promoter sequences of all *ZmSnRK* genes were obtained from the maize B73 genome database. A total of 60 *ZmSnRKs* were assessed to analyze cis-elements, including ABRE, AuxRR-core, MBS, MBSI, P-box, SARE, LTR, involving in ABA, auxin, drought-inducibility, flavonoid biosynthetic genes regulation, gibberellin, salicylic acid and low temperature response (**Figure 7**; **Table S9**). In general, 50 *ZmSnRKs* genes (90%) owned ABRE cis-elements, 22 *ZmSnRK* genes (36.7%) carried MBS cis-elements, and 28 *ZmSnRK* genes (46.7%) had LTR cis-elements. Six *ZmSnRK* genes had AuxRR-core elements, and 12 *ZmSnRK* genes had P-box. Meanwhile, only *ZmSnRK2.4* and *ZmSnRK2.12* had MBSI and SARE elements, respectively.

### Tissue-specific expression patterns of *ZmSnRKs*

**Figure 8:**
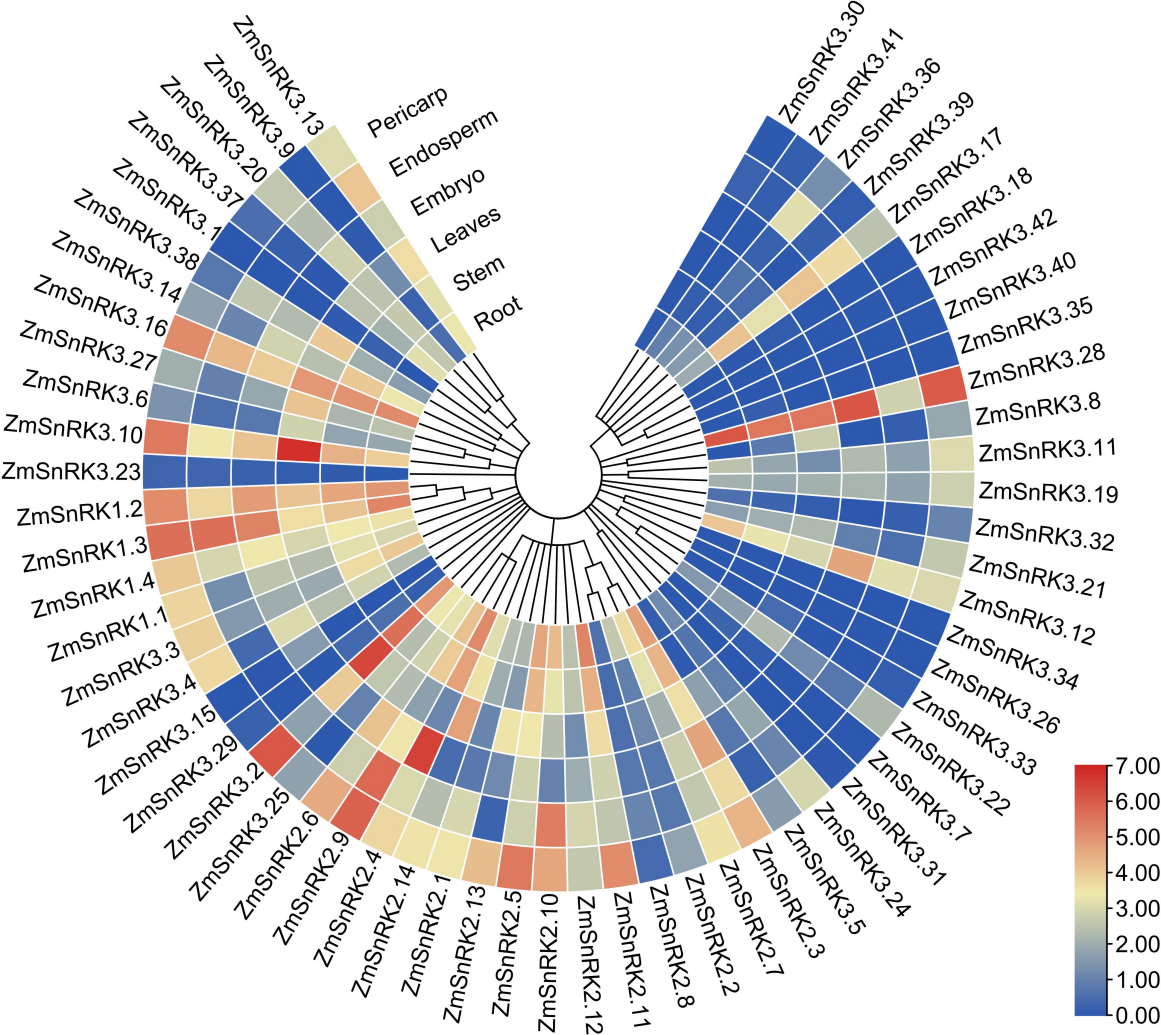
Expression profiles of the *ZmSnRK* genes in different tissues. The color scale represents expression data with row scale. Blue: low expression; red: high expression.

We compared tissue-specific expression patterns of 60 *ZmSnRK* genes in maize B73. In terms of different expression patterns, 60 *ZmSnRK* genes were classified into three groups (**Figure 8**; **Table S10**). Group 1 contains three genes (ZmSnRK3.26/3.34/3.40), and they are not expressed in all analyzed tissues. Group 2 includes 12 genes, which were expressed only in specific tissues. For instance, *ZmSnRK3.1* was expressed only in stem and leaves, and did not display expression in other tissues. Group 3 contains 45 genes expressed in all analyzed tissues. Group 3 could be classified into three subgroups. There were 14 *ZmSnRKs* belonging to subgroup 1 with high expression patterns (log2^TPM+1^>2) in all analyzed tissues, including three *ZmSnRK1s*, five *ZmSnRK2s*, and six *ZmSnRK3s*. Subgroup 2 contained only *ZmSnRK3.23* with log2^TPM+1^<0.5 in all analyzed tissues. The remaining 30 genes were assigned to subgroup 3.

### Expression patterns of *ZmSnRKs* under diverse abiotic stresses

**Figure 9:**
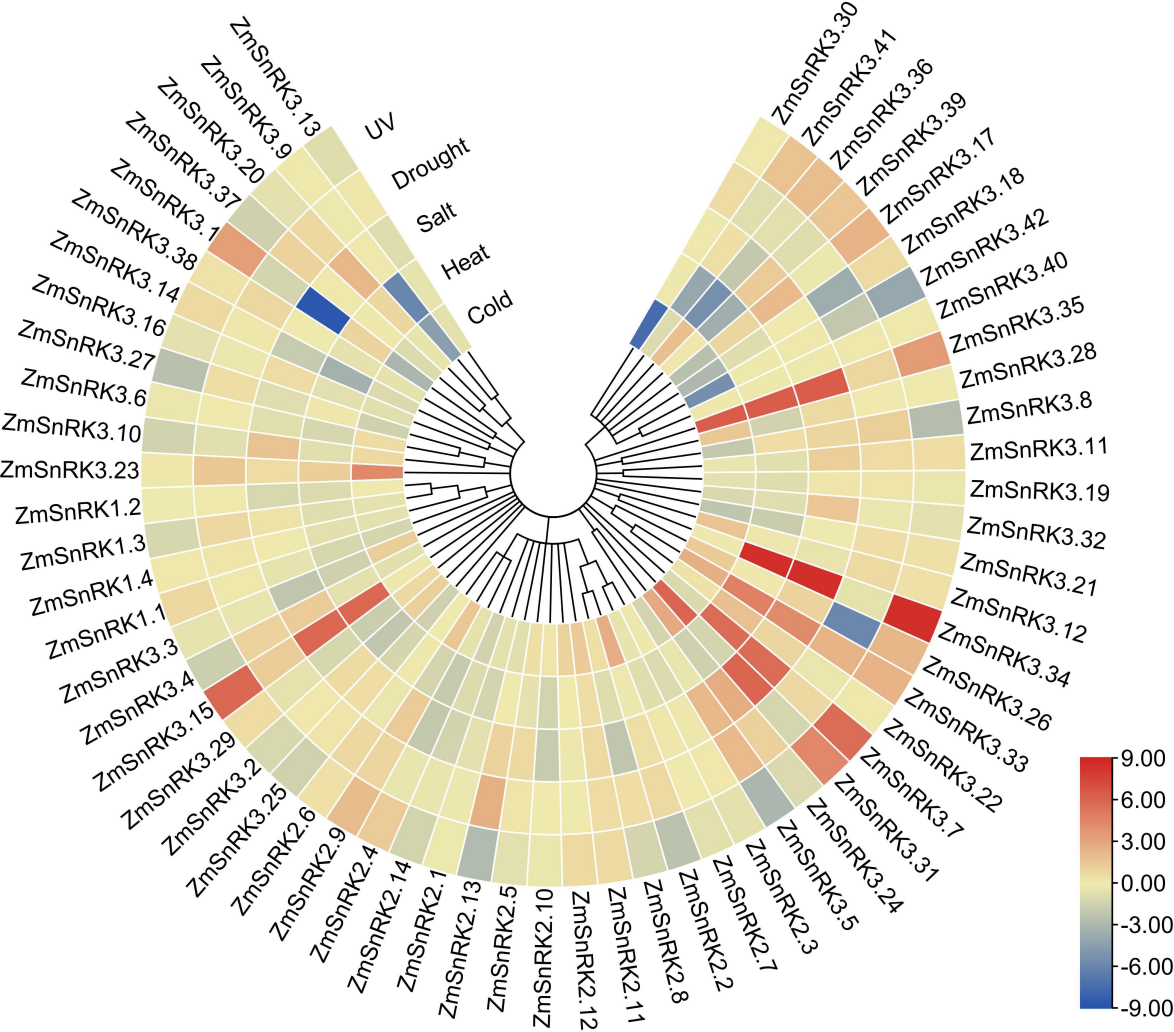
Expression profiles of the *ZmSnRK* genes under different abiotic stresses. Expression data were the ratio to control values. The color scale represents expression levels from upregulation (red) to downregulation (blue).

We collected and analyzed the transcriptome data of maize *SnRK* genes under drought, salt, high temperature, low temperature, and ultraviolet light. In general, the expression of several *ZmSnRK* genes showed significant alterations under abiotic stresses (**Figure 9**; **Table S11**). For example, *ZmSnRK2.13* exhibited up-regulation after drought stress. The expression pattern of *ZmSnRK2.2* increased under cold treatment. *ZmSnRK3.1* was down-regulated under cold and salt stress. On the contrary, some *ZmSnRKs* were not induced by any abiotic stresses listed in this study. For instance, 11 *ZmSnRK* genes showed almost no expression changes under all analyzed stresses, such as *ZmSnRK1.4, ZmSnRK2.3*, and *ZmSnRK3.13*. In addition, many genes showed opposite expression profiles in response to different abiotic stress. For example, *ZmSnRK2.2* displayed expression inhibition in UV treatment, but expression enhancement in cold stress. In addition, several *ZmSnRK* genes were chosen for qRT-PCR to verify the reliability of transcriptome data, and the results were uniform to the sequencing data (**Figure S2**, **S3**).

### Function and regulatory network of ZmSnRKs

**Figure 10:**
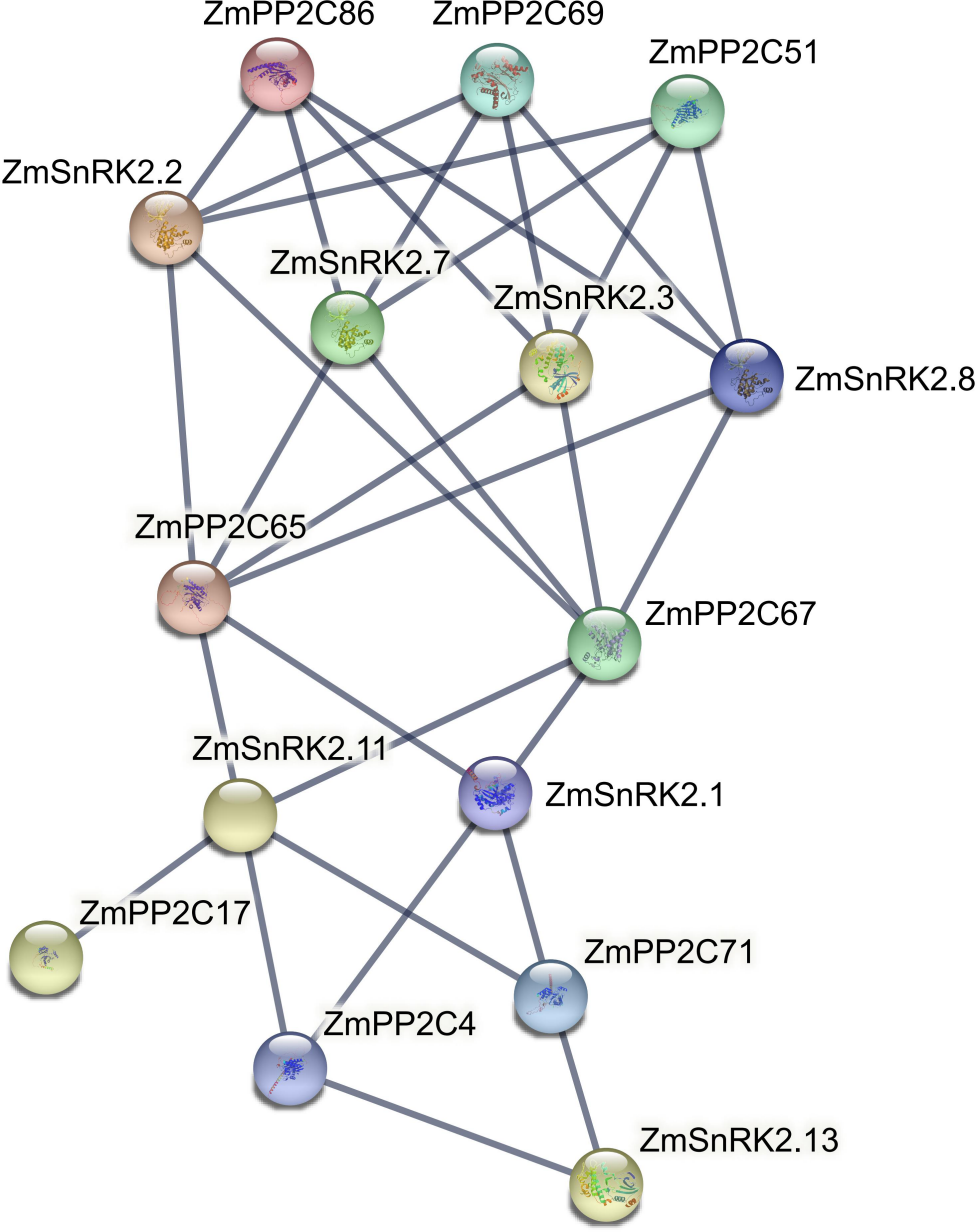
Interaction networks between ZmSnRK and ZmPP2C proteins. Information on ZmPP2Cs was shown in **Table S12**.

Protein phosphatases PP2C could dephosphorylate and inactivate plant SnRKs, the process of which prevents SnRKs from targeting ABA-dependent genes and ion channels. Hence, we used the web server of STRING to predict the protein–protein interaction (PPI) between ZmSnRKs and ZmPP2Cs to comprehend the regulatory networks of ZmSnRKs (**Figure 10**; **Table S12**). Here, eight ZmPP2Cs that could interact with ZmSnRK proteins were found. In detail, ZmPP2C65 and ZmPP2C67 could bind six ZmSnRK proteins; ZmPP2C51, ZmPP2C68, and ZmPP2C69 could bind four ZmSnRK proteins; ZmPP2C4 and ZmPP2C71 could bind three ZmSnRK proteins; PP2C17 could dephosphorylate ZmSnRK2.11 specifically. Moreover, there are also interactions within ZmSnRK proteins. For instance, ZmSnRK1.1 could bind with ZmSnRK2.14 and ZmSnRK3.12 (**Figure S1**).

**Figure 11:**
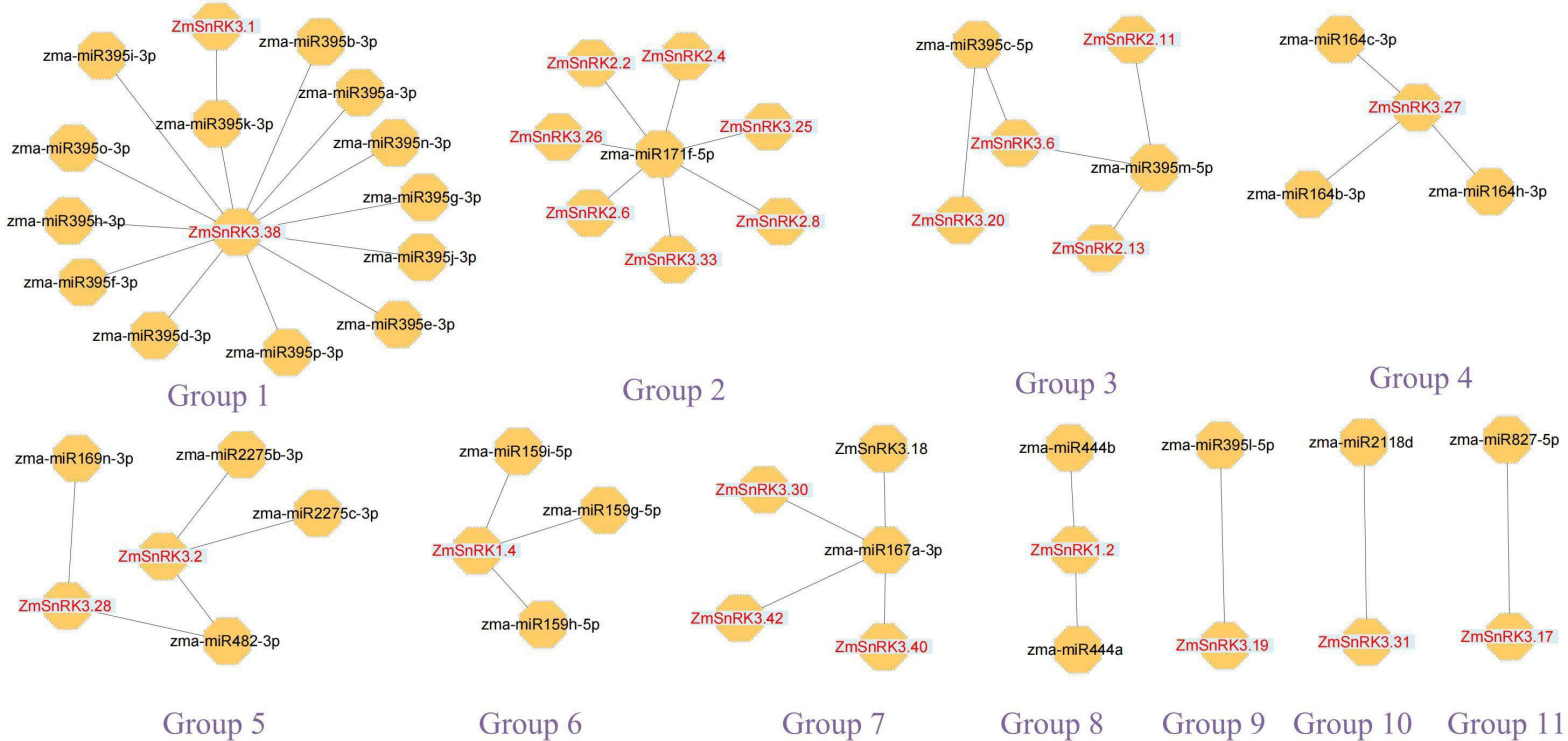
Interaction networks between miRNAs and miRNAs-acted *ZmSnRKs*. Information on miRNAs and miRNAs-acted *ZmSnRKs* was shown in **Table S13**.

We constructed the miRNA-mRNA regulatory network between 321 published miRNA in maize and 60 *ZmSnRKs*. A total of 46 putative miRNAs were identified to have a potential capacity to target and regulate 24 *ZmSnRKs* (**Figure 11**; **Table S13**). The miRNAs of *ZmSnRKs* could be grouped into 11 networks: group 1 involving two *ZmSnRKs* (*ZmSnRK3.1* and *ZmSnRK3.38*), group 2 involving seven *ZmSnRKs* (*ZmSnRK* 2.2/2.4/2.6/2.8 and *ZmSnRK* 3.26/3.33), group 3 involving four *ZmSnRKs* (*ZmSnRK* 2.11/2.13 and *ZmSnRK* 3.6/3.20), group 5 involving *ZmSnRK3.2* and *ZmSnRK3.28*, and group 7 involving *ZmSnRK* 3.30/3.40/3.42, group 4/6/8/9/10/11 involving *ZmSnRK* 3.27/1.2/3.19/3.31/3.17, respectively.

## Discussion

Protein kinases and phosphatases can recognize and transduce stress signals to diverse cellular compartments. SNF1-related kinases (SnRKs) universally exist in all eukaryotes, especially in plants. To date, the SnRK family has been identified in a number of plant species, including *Arabidopsis thaliana* (Hrabak et al., 2003), *Oryza sativa* (Kobayashi et al., 2004), *Brassica napus* (Zhu et al., 2020), and *Brachypodium distachyon* (Wang et al., 2015). However, the report concerning whole-genome analysis of the *ZmSnRK* genes has not yet been published in maize. In the current study, 60 *ZmSnRKs* have been found in the *Zea mays* genome, which were considered as *ZmSnRK1* to *ZmSnRK60* based on their chromosome distribution (**Figures 1**, **4**). Previously, *SnRK* genes were classified into three subfamilies based on different domains in C-terminus in Arabidopsis and rice (Kolukisaoglu et al., 2004). In the maize genome, 60 *ZmSnRK* genes were also classified into three subfamilies, including 4 *ZmSnRK1s*, 14 *ZmSnRK2s*, and 42 *ZmSnRK3s* (**Figures 1**, **2**; **Table S2**). Then, *ZmSnRK* genes family was systematically investigated, including evolutionary relationships, conserved motifs, subcellular localization, chromosome location, gene divergence time, promoter elements analysis, and expression patterns.

The plant SnRK1 subfamily is the key regulator of energy homeostasis (Crepin and Rolland, 2019). SnRK1 controls the activity of many enzymes and the transcription activity of several genes (Baena-González et al., 2007; Nukarinen et al., 2016), such as bZIP, ethylene insensitive3 (EIN) and indeterminate domain (IDD) (Jeong et al., 2015; Zhai et al., 2017; Muralidhara et al., 2021). For example, SnRK1 delayed senescence through phosphorylating the EIN3 transcription factor and led to its destabilization (Zhai et al., 2017). SnRK1 strongly phosphorylated bZIP63 and enhanced its dimer formation, either homodimerization or heterodimerization with other bZIPs, thus contributing to DNA binding (Mair et al., 2015). Meanwhile, Jeong et al. (2015) reported that SnRK1 was responsible for regulating various developmental processes in plants, such as hypocotyl elongation or blooming (Simon et al., 2018). In the current study, *ZmSnRK1* has four members and exhibited a high expression pattern in all tissues, but showed no alteration upon abiotic stress (**Figures 1**, **8**, **9**; **Figure S2, S3**; **Table S2, S10, S11**). Moreover, ZmSnRK1.3 and ZmSnRK1.4 were predicted to be located in mitochondria (**Table S1**). ZmSnRK1.1 and ZmSnRK1.2 were likely to be located in the nucleus and cytoplasm but with 0.657 and 0.568 reliable-index if located in mitochondria (data not shown). It suggests ZmSnRK1 may also be crucial in plant development as an energy sensor.

Plant SnRK2s, a key component of ABA signaling pathways, are regulated by ABA receptors (PYR/PYL/RCAR) and protein phosphatase 2Cs (PP2CAs). In this study, eight ZmPP2Cs were predicted to interact with seven ZmSnRK2s (**Figure 10**). It suggests that ZmPP2Cs could also dephosphorylate ZmSnRKs in maize. The SnRK2 subfamily in plants is extremely responsible for responding to abiotic stress, such as drought (Umezawa et al., 2004; Barajas-Lopez et al., 2018; Hasan et al., 2021) and salt (Zhong et al., 2020). When plants are subjected to drought stresses, they generate more ABA, thus leading to defensive stress responses to activate many SnRK2s *via* ABA-dependent or ABA-independent pathways (Hasan et al., 2022). Here, the expression level of *ZmSnRK2.13* was significantly increased after drought stress (**Figure 9**; **Figure S2**; **Table S11**), which was likely to be dephodphrylated by ZmPP2C4 and ZmPP2C71 (**Figure 10**). Moreover, the promoter of *ZmSnRK2.13* had three ABRE cis-elements and one MBS cis-element, which was associated with ABA responsiveness and drought stress (**Figure 7**; **Table S9**). One segmental duplication event was detected between *ZmSnRK2.11* and *ZmSnRK2.13* (**Figure 5**; **Table S5**), but drought exposure does not change the gene expression in *ZmSnRK2.11* (**Figure 9**; **Table S11**), indicating that *ZmSnRK2.13* was positively selected when subjected to drought stress even though it can be cleaved by the same miRNA (**Figure 11**; **Table S13**). Belda-Palazón et al. (2022) reported that SnRK2 could interact with SnRK1 and consequently cause subcellular localization changes of the SnRK1 α-subunit, the process of which are vital for repressing the target of rapamycin (TOR) and inhibiting root growth in an ABA-dependent manner. In this study, ZmSnRK2.13 could also bind to ZmSnRK1.1 and ZmSnRK2.8 (**Figure S1**). It suggested that SnRK1 could interact with SnRK2 and jointly participate in plant growth in maize. In rice, *OsSAPK8* (orthologous to *ZmSnRK2.3* and *ZmSnRK2.7*) acts as a positive regulator when plants are subjected to cold, drought, and salt stress (Zhong et al., 2020). Meanwhile, Frazier et al. (2011) reported that miR395 was up-regulated under drought stress. The current study showed that miR395 could target and cut the mRNA of *ZmSnRK2.3* (**Figure 11**; **Table S13**). *ZmSnRK2.3* and *ZmSnRK2.7*, as a duplicated gene pair, do not respond to abiotic stress (**Figures 5**, **9**; **Table S5, S11**), but showed high expression in all tested tissues (**Figure 8**; **Table S10**), indicating different mechanism in diverse species. Interestingly, *ZmSnRK2.4* have only MBSI cis-element, which is involved in flavonoid biosynthesis (**Figure 7**; **Figure S2**; **Table S9**), and its expression level was repressed under salt stress (**Figure 9**; **Table S11**). Watkins et al. (2017) found that flavonoids are involved in plant development through affecting auxin, ethylene, and ABA signaling. HSFB2b and GmMYB173 activated a number of flavonoid biosynthesis-related genes and promoted flavonoid accumulation, thus positively regulating salt tolerance (Pi et al., 2018; Bian et al., 2020). This indicates that *ZmSnRK2.4* may also be involved in flavonoid biosynthesis and regulate plant growth under salt stress.

The SnRK3 subfamily was widely recognized as CBL-interacting protein kinases (CIPKs). In plants, CBL-CIPK signaling network regulates multiple stimuli or signals (Ma et al., 2020). For example, Qiu et al. (2002) reported that AtCIPK24 could interact with AtCBL4 and activate the H^+^/Na^+^ antiporter. In the current study, *ZmSnRK3.33* showed a high expression level under five abiotic stresses (**Figure 9**; **Table S11**), which can be regulated by zma-miR171. Besides, *ZmSnRK2.2, ZmSnRK2.4, ZmSnRK2.6*, and *ZmSnRK2.8* were also cut by zma-miR171 (**Figure 11**; **Table S13**). These indicate that *ZmSnRK3* and *ZmSnRK2* may have a similar function, at least in one aspect. In soybean, GmCIPK2 acts as a positive regulator in response to drought stress in an ABA-dependent manner (Xu et al., 2021). Here, *ZmSnRK3.26* showed down-regulation with 5.9-fold changes after drought stress (**Figure 9**; **Table S11**), the promoter of which has five ABRE elements (**Figure 7**; **Table S9**). Hence, ZmSnRK3 could also respond to abiotic stresses through an ABA-dependent pathway.

In short, this research carried out a comprehensive analysis of the *SnRK* gene family in *Zea mays*. With the above results, we deduced that *ZmSnRKs* might play a pivotal role in the long-term climate resilience of maize. Numerous functional verification work is still necessary for comprehending the biological functions of *ZmSnRKs* in future research.

## Conclusion

*SnRK* genes play important roles in signaling pathways, including responses to abiotic stresses in plants. In the present study, genome-wide identification and characterization of *SnRK* genes were conducted in *Zea mays*. A total of 60 *ZmSnRK* genes were characterized and divided into three subfamilies. Phylogenetic and synteny analysis of *SnRK* genes among Arabidopsis, rice, and maize provide valuable clues for the evolutionary characteristics of the *ZmSnRK* genes. Moreover, the cis-acting elements, gene expression, and regulatory network of ZmSnRK families were also determined. These results provide insights into the functional differences, evolutionary relationships, and expression profiles of *SnRKs* in *Zea mays.*

## Data availability statement

The datasets presented in this study can be found in online repositories. The names of the repository/repositories and accession number(s) can be found in the article/**Supplementary Material**.

## Author contributions

XF and WL conceived and designed the research. XF, QM, QY, JZ, DX, and XD performed the experiments and data analyses. WL, XF, and QM wrote the article. LG and WM revised the manuscript. All authors read and approved the final article.
