# Supplementary material for: Genome-wide identification of sucrose non-fermenting-1-related protein kinase genes in maize and their responses to abiotic stresses
Source: Front Plant Sci. 2022 Dec 22;13:1087839. doi: 10.3389/fpls.2022.1087839 (PMC9815513; doi:10.3389/fpls.2022.1087839)
Supplement: Supplementary file 2 [file DataSheet_2.doc]

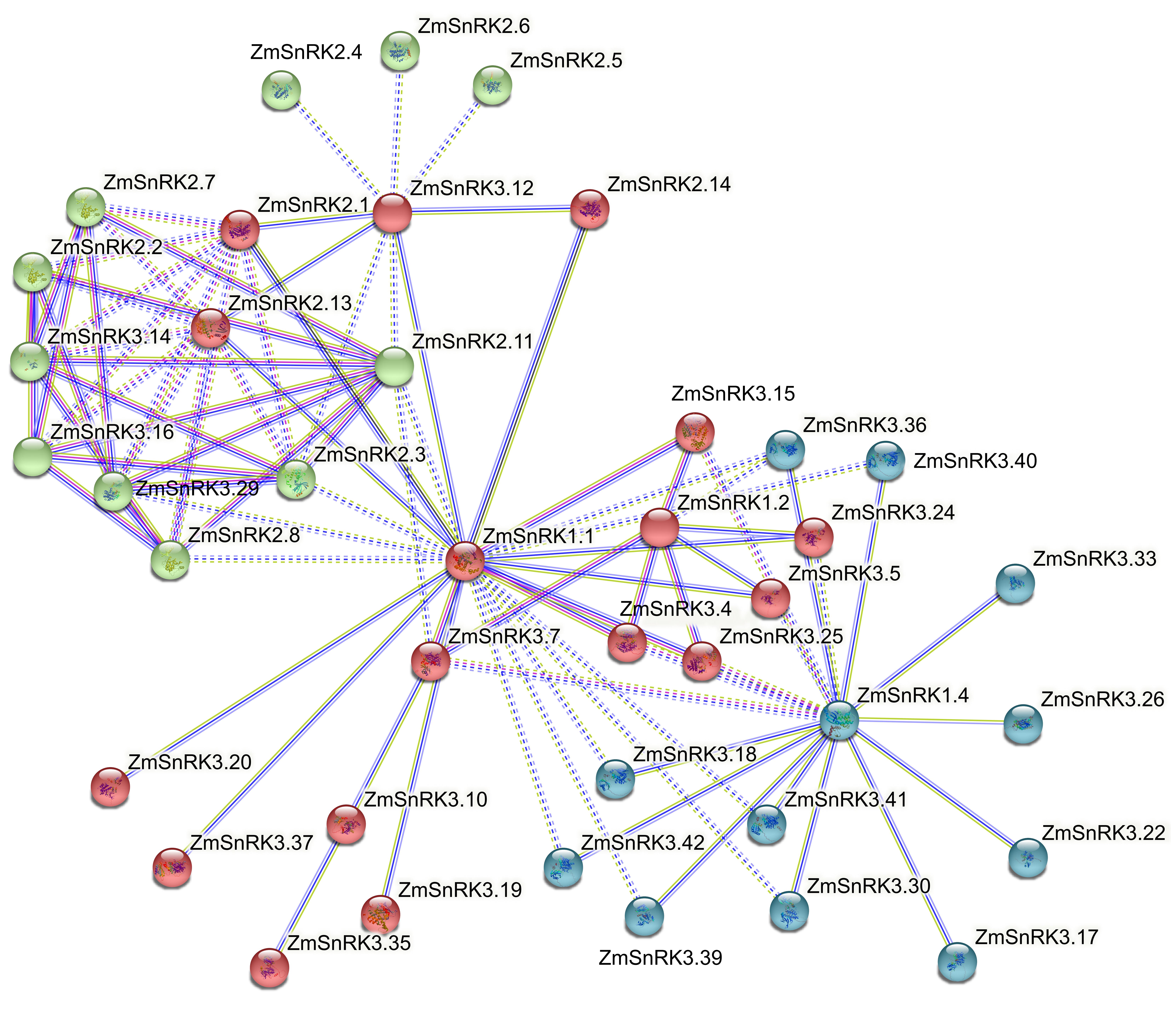


**Figure S1.** Interaction networks within ZmSnRK proteins.

**Figure S2.** Expression profiles confirmation of twelve genes by qRT-PCR in B73 under drought stress and salt stress. Roots were sampled after 24 h 10% PEG and 24h 100mM NaCl exposure in the growth cabinet. The relative transcriptional level was analyzed using 2^-ΔΔCt^ method, and the transcriptional level under control was normalized as 1.00. The experiments were performed in triplicates, and values are the mean ± SE.

**Figure S3.** Expression profiles confirmation of six genes from various tissues by qRT-PCR in B73. Roots, leaf and stem were sampled on the seventh day after transplanting. The relative transcriptional level was analyzed using 2^-ΔΔCt^ method, and the transcriptional level from root was normalized as 1.00. The experiments were performed in triplicates, and values are the mean ± SE.
